# Striving to establish a care relationship—Mission possible or impossible?—Triad encounters between patients, relatives and nurses

**DOI:** 10.1111/hex.12971

**Published:** 2019-10-06

**Authors:** Anette Johnsson, Petra Wagman, Åse Boman, Sandra Pennbrant

**Affiliations:** ^1^ Department of Health Sciences University West Trollhättan Sweden; ^2^ School of Health and Welfare Jönköping University Jönköping Sweden

**Keywords:** care relationship, ethnography, nurses, older patient, relative, triad encounter

## Abstract

**Background:**

When patients, relatives and nurses meet, they form a triad that can ensure a good care relationship. However, hospital environments are often stressful and limited time can negatively affect the care relationship, thus decreasing patient satisfaction.

**Objective:**

To explain the care relationship in triad encounters between patients, relatives and nurses at a department of medicine for older people.

**Design:**

A qualitative explorative study with an ethnographic approach guided by a sociocultural perspective.

**Method:**

Participatory observations and informal field conversations with patients, relatives and nurses were carried out from October 2015‐September 2016 and analysed together with field notes using ethnographic analysis.

**Result:**

The result identifies a process where patients, relatives and nurses use different strategies for navigating before, during and after a triad encounter. The process is based on the following categories: orienting in time and space, contributing to a care relationship and forming a new point of view.

**Conclusion:**

The result indicates that nurses, who are aware of the process and understand how to navigate between the different perspectives in triad encounters, can acknowledge both the patient's and relatives’ stories, thus facilitating their ability to understand the information provided, ensure a quality care relationship and strengthen the patient's position in the health‐care setting, therefore making the mission to establish a care relationship possible.

## 1 INTRODUCTION

The proportion of older people in the global population has increased within a short period, and this trend will continue.^1^ The increase in life expectancy accompanied by a concurrent postponement of functional limitations leads to more older persons with multiple morbidities in hospital settings.^2^ In this context, the care relationship between patients and nurses is central as it forms the basis for caring.^3^ Care relationships are characterized by a professional commitment between patients and nurses.^4^ How patients experience a care relationship may be affected by the prevailing care culture.^4^ Knowledgeable, communicative and supportive nurses are significantly related to patient perceptions of quality nursing care.^5^, ^6^, ^7^ Communication is crucial in care relationships^3^, ^8^, ^9^, ^10^, ^11^, ^12^, ^13^ for ensuring safe care that can strengthen the patient's position.^14^, ^15^ Nevertheless, dissatisfaction with communication is a common problem among patients and relatives.^16^, ^17^, ^18^ Hospital environments are often stressful and limited time can affect the care relationship.^19^ When nurses have less time, patient satisfaction may decrease.^6^

An encounter between patients, relatives and nurses can help to achieve goals and strengthen the patient's health process.^20^ Studies show that relatives are important for providing social support, reducing stress and assisting with questions.^20^, ^21^, ^22^ The importance of being informed about the care of their ill family member is crucial and relatives experienced an inadequate encounter when they were excluded from information.^23^, ^24^ Low satisfaction among relatives is related to a low level of collaboration,^25^ and poor collaboration was significantly more often associated with feelings of guilt and powerlessness in triad encounters.^26^ Previous studies have focused on decision making between physicians, relatives and patients in triad encounters^27^, ^28^ and the different roles and alliances that of those involved.^29^, ^30^, ^31^, ^32^ The care relationship between patients and nurses is also well documented^3^, ^4^, ^8^, ^33^, ^34^ but there is a gap concerning how the care relationship between older patients, their relatives and nurses in triad encounters is established. To understand different experiences of triad encounters in care relationships, it is necessary to be aware of what occurs in these encounters. Research on this issue can contribute to improvement efforts. The aim of this study was to explain the care relationship in triad encounters between patients, relatives and nurses at a department of medicine for older people.

## 2 METHODS

### 2.1 Design

The study adopted an explorative and ethnographic approach^35^, ^36^ guided by a sociocultural perspective.^37^ Ethnography describes patterns and processes in a culture or subculture.^35^ In a sociocultural perspective, experiences are socially organized. The care relationship is a phenomenon that occurs in a cultural context with social interaction, meaning that the relationship between thought, communication and action is situated.^37^

### 2.2 Setting, participants and recruitment

The setting was two wards at a medium sized public hospital in western Sweden. The wards were selected due to their uniqueness in terms of teamwork in the care of persons aged 75 years old and over with a repeated need for inpatient care. The wards were identical in terms of design, decoration and staff, and contained 24 beds each. They co‐operated regarding issues and policies. Thus, their mission and care provided was considered as similar. Patients with multiple illnesses are admitted directly to the wards without the need for a referral from the Emergency Department. On their first visit, they receive a record, in which the information is consecutively updated at each new care episode. The patient brings the record to his/her meetings with various staff in health care and municipality.

The participants were inpatients, relatives and nurses (Table 1).

The nurses were recruited at ward and nursing meetings where they received oral and written information about the study. A folder was kept on the desk in the ward so that they could hand in their consent forms at any time. The section leader informed the first author when the recruited nurses would be on duty.

A poster with information about the study was pinned on the wards’ notice boards. Every nurse was responsible for approximately eight patients on each shift. Patients with visiting relatives were contacted by the first author face‐to‐face. They received oral and written information about the study and were invited to participate. All invited patients and relatives agreed to participate and gave written consent. The patients were treated for chronic diseases, for example heart failure and respiratory problems. Patients identified by the nurse in charge as critically ill were excluded, for ethical reason. The participating relatives were husbands, wives, daughters, sons, sons or daughters in law, friends and grandchildren.

**Table 1:** Participating patients, relatives and nurses

	Number	Age, range (mean)	Male	Female	Working year at the ward
Patients	21	77‐96 (4287)	8	13	
Relatives	21	30‐90 (8759)	7	14	
Nurses	19	23‐62 (42)	0	19	0,5‐7

### 2.3 Data collection

Data collection took place from October 2015 to September 2016 and involved audio‐recorded communication from participatory observations of naturally occurring triad encounters and informal field conversations as well as field notes. Participatory observations enable one to see the interaction and hear the communication between those in the meeting, while the informal conversations increase understanding of the context,^35^ and thus, they were considered suitable methods for explaining the care relationship in triad encounters.

The participatory observations^35^ (n = 21) covered 110 hours of audio‐recorded communication material and took place at different times, days and locations (eg patient rooms or meeting rooms) to obtain a complete picture as possible.^38^ No patient or relative participated in more than one observation, but two nurses participated in two observations. Every observation lasted for 30‐90 minutes. The field notes with reflections were manually written during/after the observation and transcribed after the participatory observation.^35^

Directly after the triad encounter, informal field conversations were held with each patient, relative and nurse (n = 63), which lasted for 10‐15 minutes, and were audio‐recorded. They followed what had occurred during the triad encounter using open questions^35^, ^36^, ^37^, ^38^, ^39^ like ‘*can you tell me about the previous encounter, and how you experienced it? What did you talk about? Why?’* followed by questions; ‘*Did you mean…when you said…? Can you explain?’* Data were summarized to give the participants a chance to make further comments.

### 2.4 Data analysis

The recordings of the communication during the participatory observations and informal field conversations were transcribed verbatim. The texts were repeatedly read to obtain a sense of the whole and compared with the field notes.^35^ The data were then read word by word and reflected on, after which the derived meaning units were manually transferred to coding sheets. Codes were sorted into categories of meaningful units, which were examined for patterns that explained the phenomenon of interest.

Then, the data were analysed again with focus on the perspective of each party. These perspectives were matched with the categories and broken down into smaller concepts that distinguished specific characteristics of each perspective. Finally, the entire data were read as a whole and a process identified. The analysis resulted in three categories and six sub‐categories. During the analysis process, all data and emerging categories and sub‐categories were considered and discussed by all authors until agreement was reached and an overall theme formulated. The result is presented together with quotations, labelled with the number of the encounter, for example nurse 21 means the nurse in the 21st observation, while field note 6 indicates the notes made during the 6th observation.

The consolidated criteria for reporting qualitative research (COREQ)^40^ guidelines have been followed.

## 3 RESULTS

**Figure 1:**
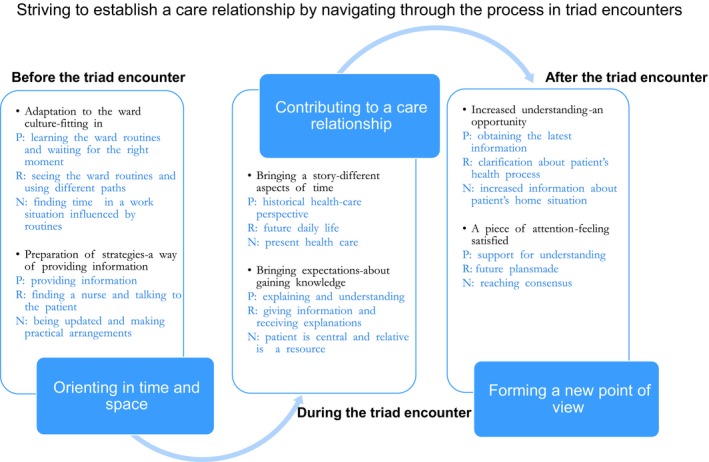
Patients (P), relatives (R) and nurses (N) navigating through the process before, during and after triad encounter

The result reveals a process where patients, relatives and nurses strive to establish a care relationship using different perspectives and strategies for navigating before, during and after a triad encounter. The process comprises the following categories: orienting in time and space, contributing to a care relationship and forming a new point of view (Figure 1).

### 3.1 Orienting in time and space

This category explains the participants’ ways of adapting to the ward culture and preparing strategies before the triad encounter to enable a care relationship.

#### 3.1.1 Adapting to the ward culture‐ fitting in

Adapting to the ward culture in this context means that the patients rapidly learned the norms and values by becoming familiar with activities that they thought were important, activities such as ward routines and nurses’ schedules. They adapted to routines because they understood that such activities maximized ward efficiency and believed that they could obtain help more easily if they were adapted. The patients described different activities, such as meals, medical treatment and daily routines when nurses visit all the patients.

The door of the patient’s room is open. The patient is just starting to eat her supper. She has diabetes and is waiting for the nurse to come with the insulin injection. The patient sits in bed and talks to her relative but becomes silent as soon as she sees the nurse entering the room. The nurse greets the patient by waving her hand and smiling. The patient looks at her wristwatch and nods. Her relative looks confused and whispers: ‘what*?’* The patient whispers back: ‘they [nurses] *are so clever. They come several times a day and visit me, at least three I think, yes routines you know’.* The nurse arrives at the bed, smiling, nodding and points at the food saying: ‘*I see that you have already started, here comes the insulin*’. [Field note 8]

The relatives understood the importance to fit in and of following routines for obtaining information and used different strategies for arranging a meeting with a nurse such as phoning to make an appointment or seeking a nurse during visiting hours. They also found out the nurses’ routines, for example when the nurses introduced themselves at the start of a new shift. The relatives learned to watch out for the right moment to catch the nurses.

They tell us everything. This brief meeting was only with the nurse who walked around and introduced herself. I have noticed that they usually do it at this time. You must catch the right moment. [Relative 21]

The nurses’ work schedules were often filled with routines and they considered all activities as part of their duties. During the informal field conversations, the nurses expressed things they usually did or what they thought was best for the ward. They were anxious to find time and space to meet patients and relatives, stating that they worked hard to be flexible and make everyone happy.

Well, that you visit all the patients, make a small check. I introduce myself, inform about my working hours and then ask how they are. There are different things I need to check. [Nurse 9]

#### 3.1.2 Preparation of strategies—a way of providing information

Before the triad encounters, the patients prepared strategies to provide information and ensure the right nurses received it. Some had a notebook containing information, while others wrote notes and gave them to the nurses. Some also prepared information for their relatives to pass on to the nurse. These strategies made the patients feel prepared to providing information.

I think they [nurses] are good, 99% are good. They are good if they just stand still. Yes, sometimes they just run away. They are needed elsewhere and do not have time to finish the chat. I have written, (becomes silent and shows a piece of paper with notes on it) and the important details are marked, so I am prepared when they come. [Patient 1]

The relatives prepared themselves by learning whom to talk to and where to find them. They tried to find a person with a nurse nameplate, which was difficult sometimes due to small lettering or no nameplate at all, which led to misunderstandings. In some cases, they guessed it was the nurse because it was the right time in the schedule. The relatives also prepared themselves by talking to the patient and asking if there was anything they should mention to the nurse.

Well, I may take over the role. Now that I see that she [patient] is unable to say what she wants herself I cannot ignore the responsibility. I'm her daughter. She needs my help. [Relative 12]

Nurses prepared themselves by reading the patient's journal and sometimes wrote notes that they kept in their pocket. They also tried to find and reserve time for meetings. Most preparation time was spent updating themselves and making practical arrangements such as finding a quiet place to sit and communicate.

The nurse is planning to visit the patient in her room and prepares herself by carefully reading the patient’s journal and writing down facts on a piece of paper. She reads through the note once more and then puts it in her pocket. The nurse closes the door when she enters the room. The patient is sitting on the bed. She has an oxygen mask over her face and breathes heavily when talking. She looks pale. The relative is sitting on a chair, next to the bed. He looks up when the nurse arrives and nods in the nurse’s direction. The nurse goes into the room and stops next to the patient. She puts her hand in her pocket but does not take out her note. She greets by saying hello, how are you today? [Field note 6]

### 3.2 Contributing to a care relationship

Nurses, relatives and patients have a mission to perform during the triad encounters. The mission is to establish a care relationship in such a way that co‐operation between all parties is continuous and that they all have something to contribute. This category explains how the participants focused on different time aspects during the encounter and how all bring their own expectations to the situation.

#### 3.2.1 Bringing a story—different aspects of time

The patients wanted to tell the nurse the whole story, from the start of their illness until admission to the ward. Their focus was based on a historical health‐care perspective. Many had hearing or perceptual difficulties and expressed that the nurses constantly moved when they were speaking, which made it hard to follow the conversation. However, when the nurses listened, the patients experienced they could bring their story.

She [nurse] gives time to talk and ask questions, not bothered in any way, even though she is busy, I suppose, but does not show it. She listens. She really did see me. She did not hurry. My situation is complicated. I’ve got several problems you know but today I could think and ask… [Patient 21]

During the triad encounter, relatives obtained information about the patient's health status, test results or planned examinations as the patient's representative. Relatives saw the communication situation as an occasion where they could support and help the patient's recovery. They planned for the future when the patient returned home, which could include the patient's needs, support and resources.

They [nurses] are kind and talk about the situation and the health status, we made plans, the nurse says that he is struggling on. That’s fine. I needed to know more about when he should take his medicine and if he will get any more help at home. Yes, that sort of information. Otherwise you never know how long he will be in hospital. [Relative 5]

Nurses focused on the present situation. They never knew what topic would come up during the encounter, but by asking and listening, they tried to gain increased understanding of the patient's life situation. The learning atmosphere was created by the use of didactic questions (how, what, why, when) to make the situation clearer. They also did their best to mediate calmness and learned how to handle different situations by experience.

One has many conversations, like how everything is or what to do and why. Often family members are here. They are visiting, and usually have questions and I also have questions for them. I think it’s important to let them ask their questions. I do not want to forget anything. Well, I informed them about why she got the infusion, why she got it, but sometimes it is difficult. Eh, I mean, it is good to have a relative there too. Then they can both can listen and ask questions. [Nurse 7]

#### 3.2.2 Bringing expectations—about gaining knowledge

The expectation that the patients brought into the triad encounter was to meet someone who could explain what happened during the care process. Time for reflection gave them an opportunity to understand their illness. Sometimes they experienced that they were not a part of the care relationship, for example when the nurse only directed information to the relative or was interrupted by the telephone. They therefore tried to fit in and be pleasant, in order to obtain information about their illness. Patients also described trying to be nice, polite and answer the nurse's questions, which they thought they were expected to do.

Patient: Well, I joke with them, one must try to make life a bit more fun and, after all, the conversation is quite health related. You must be pleasant. The information that the nurses give me is important. [Patient 21]

Relatives’ expectations during the triad encounters concerned giving and receiving care information. They expressed that the encounter was a forum for questions, answers and listening that increased understanding about the care. Sometimes they felt ignored and that the nurses did not take them seriously, which was not consistent with their expectations. Understanding the patient's illness was important for them and they usually spoke as the patient's advocate.

Relative: It's important, as a relative, that I feel they [nurses] are listening to us and we can all ask questions, not be ignored. My expectation today was to receive some information about my mother, and that was fulfilled. I also had some information to give, but my mother is well enough to say what she wants, she does not forget. [Relative 13]

Nurses expected the patients to be in focus during the triad encounters and believed it was important to involve them. They turned to the patient while talking, were friendly, cheered the patient up, gave her/him notes and repeated information. The nurses had experiences and expectations of relatives as a resource, not only for the patient but for themselves. Nurses often gave relatives instructions before the patient's return home, so that they could be supportive and know what to do if questions arose. Relatives were seen as a link between the patient and the nurse because they know the patient and are familiar with her/his reactions and body language.

Nurse: Relatives can be a great resource for informing about what happened, what the home situation is like today and for forward planning. They can also be a huge support to the patient. [Nurse 21]

### 3.3 Forming a new point of view

After the triad encounter, all parties involved had formed a new perspective on the care situation, even if the quality of the communication in the encounter varied and whether or not the mission was experienced as completed. The participants received an increased understanding, which opened up a new perspective when they all got some attention.

#### 3.3.1 Increased understanding—an opportunity

When patients received information, had a chance to ask questions and receive answers, it gave them an opportunity to understand their own illness and care. Sometimes they did not understand all the information, because of disabilities or the fact that they were not spoken to. On these occasions, they trusted in their relative to give them more information. The patients gained a new point of view when they got the chance to tell their own story and learn about their hospital stay.

They [nurses] are so skilled. They explained to me about the medications and answered my questions about how I should take the medicine. Now I know how to take the medicine and understand why. [Patient 2]

The relatives gained increased understanding of the patient's health situation. They were worried about the patient and when not satisfied with the information, they became frustrated and tried to find a different nurse to explain things or phoned the ward. In this way, they learned about the patient's care.

Relative: I got answers to what I asked about, what I was thinking about. I got everything explained. The nurse made it clear to me. He [patient] has been there for a long time. He lives at a retirement home. Then he was at another ward where he got physiotherapy. Yeah, he was dehydrated. That's why he came to this department. Oh, he was so sick. You cannot imagine. [Relative 16]

After the triad encounter, the nurses’ understanding of the patient's situation increased and they learned about her/his home situation, interests and understanding of the illness. They expressed that the best communication was when all parties involved could share information with each other. At such times, the nurses had a sense of being on the same level. Although there were also situations when the nurses were not satisfied, they always made sure they got the necessary information from the patient and/or relative.

The response is important. I could feel that we all had something to share. Well, all of us, it felt like we were on the same level in the conversation, not only me talking. We were sharing a moment. [Nurse 13]

#### 3.3.2 A piece of some attention—feeling satisfied

After the triad encounter, the patients experienced satisfaction because of the attention they received, which increased their understanding of the health situation. They also had an uplifting experience of the nurse's personal touch, friendly tone and positive demeanour. The patients were grateful after the encounter and even when it was experienced as less positive, they were satisfied because of the attention they had received.

Everything went well. They are so nice and pleasant. I think that's really fine. Absolutely! Definitely! What can I say? Great support? [Patient 9]

Relatives expressed a sense of receiving attention when the encounter was arranged. It was often difficult to decide on a time to meet and they sometimes needed to repeat their request. They expressed satisfaction when they understood the patient's problem and future plans.

Yes, the nurse spoke in a very objective and thoughtful way. Forward, yes, about the future. When my husband comes home, about what to do and how to do things. [Relative 9]

The nurses had different meetings with patients and relatives during their shift. Sometimes the meeting failed but they experienced, that even an unsuccessful meeting could be rectified. In addition to responding to questions, they gave and received attention, by posing questions, to patients and relatives to ensure that they had the right information. When the communication in the encounter went well, the nurses felt they all reached agreement and that the mission had been possible.

Nurse: I will insert a new medicine list in your portfolio cover [Nods].

Patient: Good [Smile]. That’s fine.

Relative: Yes, thank you.

Nurse: Doesn’t he have a portfolio?

Relative: Yes, he does.

Nurse: Fine. Then I will write everything that we agreed on, in it. Okay?

Patient: Good [Nods]

Relative: Is there a medicine list and everything there?

Nurse: Absolutely. A new medicine list is there, but I can phone you tomorrow when he comes home. Is that all right?

Relative: Yes, that will be fine (Transcribed communication related to patient, relative and nurse 7).

## 4 DISCUSSION

The aim of the study was to explain the care relationship in triad encounters between patients, relatives and nurses at a department of medicine for older people. The findings reveal a process where patients, relatives and nurses use different strategies for navigating before, during and after an encounter and how the ward culture, preparation, time aspects, expectations, understanding, and attention influence and affect the process. Nurses’ awareness of this process can facilitate a care relationship and understanding for all involved. The context influences the structures that shape different activities, as they are all part and product of their particular social context where experiences are created together.^37^, ^41^ Norms, values and structural activities can both enable and prevent a meeting.^42^ The social and cultural context, as well as interpersonal competence, are important in the care relationship. Nurses^42^ need to take cognizance of these factors when establishing a care relationship. Working with staff values and beliefs is a crucial first step in developing practice and affecting cultural change.^43^

The patients rapidly learned to fit in and adapt to the structural activities. It has been shown that patients who do not adapt to the ward routines and culture are classified as troublesome.^44^ There is then a risk that the care culture can lead to suffering for the patient.^45^ For the relatives, on the other hand, it was sometimes difficult to figure out who was who among the staff, which led to misunderstandings. Both patients and relatives have difficulty in differentiating between the various health‐care professionals providing care.^19^ Therefore, it is important for nurses to make sure that they are recognized by having a professional presentation and distinguishable nameplates.

The patients prepared their relatives to help them and asked them to communicate with the nurse on their behalf. From a patient point of view, these findings are similar to the results of recently published research,^46^ where older patients wanted to participate in conversations, but when experiencing difficulties, they employed strategies to gain a position of influence by asking their relatives to help with communication about needs and care.^21^, ^47^

The result shows that in triad encounters, those involved focus on different perspectives. The meeting is perceived as a mission that is possible if nurses understand how to navigate between the different perspectives and acknowledge both patients and relatives in their stories, thus leading to increased collaboration. This is consistent with the earlier findings,^5^ where patient experiences of the care relationship, person‐centeredness, respect and the strive to make the whole person visible, where she/he is seen and met as a person,^48^, ^49^ seem to play a major role in patients’ perception of quality nursing care. Among relatives, feelings of guilt and powerlessness are common, which are associated with poor collaboration.^26^ Their satisfaction increased when they were enabled to participate and collaborate with the nurses and patient.^25^

All three parties bring different expectations into the triad encounters, and patients’ expectation was to be able to ask questions. The most common barrier is that older patients perceive having no opportunity to ask questions when they have difficulty understanding.^50^ The participating parts take different roles in the encounter^28^, ^29^ but the attention of the patients’ need is necessary.^45^ Although it may seem obvious that nurses should be aware of and listen to both patients and relatives, communication can fail in a stressful environment.^51^ Communication is part of nursing activities and small talk can be a resource for achieving a nursing goal, normalizing unpleasant procedures or conveying sensitive information.^52^ Patients, relatives and even nurses need some attention, and this study shows that the wards constitute a stressful environment with a heavy workload for nurses. If suffering related to care occurs, it may cause the patient's health process to deteriorate.^3^

In the specific culture in which the triad encounters between patient, relative and nurse take place, all participants must contribute to understanding and learning, which underlines the complexity involved. It is up to all the participants to ensure that the mission to establish a care relationship is possible. They all bring their own expectations and perspectives and have a responsibility to make efforts to participate in the mission to establish a care relationship.

### 4.1 Trustworthiness and limitation

Participatory observation, field notes and informal field conversations made it possible to examine data relating to the same situation from several different perspectives. Comparison of the different data collection techniques also constitutes a basis for checking interpretations.^53^ Trustworthiness is strengthened by the informal field conversations directly connected with the triad encounters, which meant that the participants had a clear memory of the communication event.^35^, ^39^ With regard to the study's credibility, detailed descriptions of the method, participants, setting, data collection and results have been provided.

During participatory observation, there is a risk of the author's presence affecting the situation in that the observed party may modify her/his behaviour in response to the knowledge of being observed.^35^ Patients and relatives are in a dependency situation, and the nurse may make some extra effort. To reduce this risk, the author was present at the ward for two weeks before the study started, to get to know the nurses and environment. Reflective notes about the researcher role were regularly written during and after sessions in the field.^54^

Previous experiences may influence thoughts and perceptions about a phenomenon.^39^ In this study, the research team was cross‐professional, and the different preunderstanding was reflected on. The analysis was critically discussed by all the members of the research team and a careful description of the steps, and how the analysis was conducted was formulated. Quotations have been provided to strengthen confirmability.^39^, ^54^

A limitation of the study is the collection of data from only two locations. The findings are therefore more appropriate for achieving conceptual understanding than for generalization. Another limitation is that the transcripts were not returned to all participants for comment or correction. However, during the interview, the participant's statements were summarized so that she/he could confirm, correct or clarify, as appropriate. The results were also presented to two nurses who confirmed them.

## 5 CONCLUSION

The result indicates that adaption to the ward culture, preparation, time aspects, expectations, understanding and attention are crucial for patients, relatives and nurses striving to establish a care relationship. The knowledge of how to navigate through the process before, during and after triad encounters can help nurses to understand their own perspectives, as well as those of patients and relatives. Awareness of this process may enhance the nurses’ understanding of the complexity involved in obtaining and providing the information necessary to build trust and create a quality care relationship, as well as strengthen the patient's position and make the mission possible.

### 5.1 Relevance to clinical practice

The results emphasize the importance of awareness that the triad encounter involves a process where all parties have different perspectives. This knowledge means that nurses will be better prepared to use a holistic approach to improve patients’ and relatives’ understanding of the information.

The findings can be reflected on by nurses in team and clinical training interventions to increase awareness of care relationships and improve patient‐relative‐nurse communication in triad encounters. In a wider perspective, the knowledge can be used in the context of nursing education, especially with regard to students’ clinical placement, orientation and communication with patients and relatives.

## CONFLICT OF INTEREST

The authors declare that there is no conflict of interest.

## AUTHOR CONTRIBUTIONS

All authors (AJ, ÅB, PW, SP) made substantial contributions to the study's conception and design. AJ acquired that data and all of the authors (AJ, ÅB, PW, SP) participated in data analysis and interpretation. All of the authors made substantial contributions to the drafting of the article or revised it critically for important intellectual content.

All of the authors agreed on the article's final version and meet at least one of the following criteria [recommended by the ICMJE (http://www.icmje.org/ethical_1author.html)]:

- substantial contribution to conception and design, acquisition of data or analysis and interpretation of data;
- drafting the article or revising it critically for important intellectual content.

## ETHICAL CONSIDERATIONS

The study was approved by the regional ethical review board (Dnr: 584‐15). In accordance with the Declaration of Helsinki,^41^ verbal and written information was given to participants about the voluntariness, risks and benefits of participation, that they could withdraw at any time and that the results would be presented in a manner that would safeguard their identity. All participants gave informed consent. All confidential information was stored in a secure place to prevent unauthorized persons accessing it.

## AUTHOR BIOGRAPHIES

**Anette Johnsson** (RN, RNT) is a PhD student in Healthcare Sciences at the Department of Health Sciences, University West, Trollhättan, Sweden, and at the School of Health and Welfare, Jönköping University, Sweden.

**Petra Wagman** (PhD, Reg. OT) is Associate Professor in Occupational Therapy at the Department of Rehabilitation, School of Health and Welfare, Jönköping University, Sweden.

**Åse Boman** (RN, RNT, PhD) is a senior Lecturer in Healthcare Sciences at the Department of Health Sciences, University West, Trollhättan, Sweden.

**Sandra Pennbrant** (RN, RNT, PhD) is Associate Professor in Healthcare Sciences at the Department of Health Sciences, University West, Trollhättan, Sweden.

## Data Availability

The data that support the findings of this study are available on request from the corresponding author. The data are not publicly available due to privacy or ethical restrictions.
